# Amoxycillin and clavulanic acid induced Stevens-Johnson syndrome: A case report

**DOI:** 10.17179/excli2017-345

**Published:** 2017-05-18

**Authors:** Maheen Zaidi, Syeda Kashaf Zaidi, Moomal Bhutto, Mohammad Yasir Umer

**Affiliations:** 1 Jinnah Sindh Medical University, Karachi, Pakistan; 2 Dow University of Health Sciences, Karachi, Pakistan; 3 Jinnah Postgraduate Medical Centre, Karachi, Pakistan; 4 Baqai Medical University, Karachi, Pakistan

**Keywords:** Stevens-Johnson syndrome, amoxycillin and clavulanic acid, hypersensitivity, lesions, Naranjo Adverse Drug Reaction Probability scale

## Abstract

Stevens-Johnson syndrome (SJS) is an immune mediated hypersensitivity reaction. Significant involvement of oral, nasal, eye, vaginal, urethral, GI and lower respiratory tract mucous membrane may develop. It is usually a reaction due to a medication or due to an infection. In 95 % of case reports, drugs were found to be an important cause for the development of SJS. In this case report, a 32 year old female reported chief complaint of itch skin eruptions all over the body along with erosive lesions on tongue, lips, buccal mucosa and genital mucosa. The reaction occurred after administration of augmentin (containing amoxycillin and clavulanic acid). She was treated with antimicrobials, antiallergics and conservative management. The patient improved and was discharged from the hospital. Causality assessment using Naranjo Adverse Drug Reaction Probability Scale revealed that amoxycillin and clavulanic acid combination was a possible cause for the adverse reaction with a score of 4.

## Introduction

Stevens-Johnson syndrome (SJS) is a rare but very serious disorder of skin and mucous membranes. It is usually a reaction due to a medication or due to an infection. It is considered as an acute life-threatening condition and a medical emergency and requires hospitalization. In SJS recovery takes weeks to months, depending on the severity of the patient's condition (Mayo Clinic, 2014[12]). The proportion of females has been estimated to be 33-62 %. The largest series reports 39.9 % of females in a group of 315 patients with Stevens-Johnson syndrome.

In a large cohort, the mean age of patients with Stevens-Johnson syndrome was 25 years. In a smaller series, the mean age of patients with Stevens-Johnson syndrome was reported as 47 years. However, cases have been reported in children as young as 3 months and adults as old as 78 years (Foster, 2016[7]).

SJS may present as a nonspecific febrile illness leading to malaise, headache, cough, rhinorrhea with polymorphic lesions of the skin and mucous membrane characterized by acute blisters and erosions (French, 2006[8]). Various etiologic factors have been implicated as causes of Stevens-Johnson syndrome. Drugs most commonly are blamed. The four etiologic categories are infectious, drug induced, malignancy-related and idiopathic. Stevens-Johnson syndrome is idiopathic in 25-50 % of cases. Drugs and malignancies are most often implicated as the etiology in adults and elderly persons. Pediatric cases are related more often to infections (Foster, 2016[7]).

As per case reports and studies, more than 100 drugs have been identified as causes of SJS (Schöpf et al., 1991[18]; Roujeau and Stern, 1994[15]). The drugs that cause SJS commonly are antibacterials (sulfonamides), anticonvulsants (phenytoin, phenobarbital, and carbamazepine), nonsteroidal anti-inflammatory drugs (oxicam derivatives), and oxide inhibitors (allopurinol) (Deore et al., 2014[4]).

## Case Report

A 32 year old female came to our hospital in emergency department with itchy skin eruptions all over the body. She gave a history of sore throat for which she was administered augmentin (containing amoxycillin and clavulanic acid). The eruptions were seen after taking first dose which she took at night. The eruptions were erythematous, hyper pigmented, target-like, round lesions. The eruptions were 2-3 cm in diameter and present throughout the body, more concentrated on the upper and lower limbs, upper chest, abdomen, face, palms and soles. On examination, erosive lesions were present on the lips, buccal mucosa and the tongue. Painful erosions were also seen on the genital mucosa. Redness of eyes and blurring of vision was also noted. A diagnosis of Stevens-Johnson syndrome was made. Subsequently, multiple vesicles and bullae with antral necrosis developed in the area of the lesions. Blistering of the lesions was noted. Swelling of the face and lips were noted. A slough and whitish plaques were observed over the tongue. The lesions later crusted on the skin and the oral cavity. Bleeding time and clotting time were in the normal limits. Absolute eosinophil count was normal. Blood urea and serum creatinine levels were normal. Liver function tests were normal. Neutrophils were increased.

The following drugs were administered:

- Injection piperacillin/tazobactam 4.5 g i.v. three times a day
- Injection linezolid 600 mg i.v. two times a day
- Injection albumin 50 ml i.v. once a day for three days
- Injection pheniramine 8 mg i.v. twice a day
- Tablet fluconazole 150 mg once weekly
- Tablet loratidine 10 mg at bed time daily
- Tablet folic acid 5 mg once daily
- Syrup paracetamol two teaspoons full three times a day
- Syrup K-lyte (potassium bicarbonate potassium citrate) two teaspoon full three times a day
- Potassium permanganate mouth wash
- Polymyxin B ointment
- Fusidic cream
- Nystatin drops
- Saline wash.

During her stay in ward swelling was noted in her both legs and diagnosis of DVT was made through color Doppler. Following drugs were given for DVT:

- Tablet Rivaroxaban 15 mg twice a day used for seven days, then shifted to Tablet warfarin 10 mg once a day
- Injection Enoxaparin sodium 60 mg subcutaneously twice a day.

The patient improved with the above treatment and was subsequently discharged from the hospital with the advice not to be administered beta-lactam antimicrobials in the future.

## Discussion

In 1922, Stevens and Johnson described 2 male patients of 7 and 8 years old, who developed extraordinary generalized eruption with fever and inflamed buccal mucosa (Barvaliya et al., 2011[3]; Stevens and Johnson, 1922[19]). SJS can be differentiated from other skin conditions on three clinical criteria, (i) the pattern of individual skin lesions, (ii) distribution of lesions, and (iii) extent of epidermal detachment. The characteristic findings in SJS are widespread erythematous or purpuric macules which form flat atypical target lesions as the disease progresses to cause full thickness epithelial necrosis (French, 2006[8]).

In the oral cavity, SJS causes widespread ulcerative lesions. A prodrome occurs in about 30 % of cases and may begin within 1 to 3 weeks of starting a new drug and lasts 1 to 2 weeks before the onset of mucocutaneous manifestations, presenting with flu-like symptoms, sore throat, headache, arthralgias, myalgias, fever, bullous and other rashes, pneumonia, nephritis or myocarditis (Farthing et al., 2005[6]). Balanitis, urethritis and vulval ulcers may occur. Our patient did not report any prodrome, but skin, mouth and genital ulcerations were present. Drug-induced SJS is characterized by mucosal erosions plus widespread distribution of atypical targets or purpuric macules and epithelial detachment involving less than 10 % BSA on the trunk, face and extremities (Ayangco and Rogers, 2003[2]). SJS has to be clinically differentiated from viral stomatitides, pemphigus, EM, TEN and the sub-epithelial immune blistering disorders like pemphigoid. There are no specific diagnostic tests for SJS (Farthing et al., 2005[6]). Our case showed ulceration of oral cavity, involvement of eye with redness, ulceration of genital region along with numerous healed lesions on chest, abdomen and limbs which showed typical appearance of “target lesions”. The lesions were widespread as compared to EM, which is localized.

Adverse drug reactions (ADRs) are one of the leading causes of death among hospitalized patients and occur in 0.3 to 7 per cent of all hospital admissions. These may vary from mild rashes to severe reactions such as Stevens-Johnson syndrome (Doshi et al., 2012[5]). Hällgren et al. (2003[11]) stated that antibiotics are the most common cause of Stevens-Johnson syndrome, followed by analgesics, cough and cold medication, NSAIDs, psychoepileptics and antigout drugs. Of antibiotics, penicillins and sulfa drugs are prominent; ciprofloxacin has also been reported (Hällgren et al., 2003[11]). Patel et al. (2012[13]) reported in their study that antimicrobials were the most commonly suspected drugs (45 %) causing SJS as has also been reported in Australia (Sanmarkan et al., 2011[17]). In a study conducted on 225 references in India, 10 references were included as per selection criteria. The major causative drugs were antimicrobials (37.27 %), anti-epileptics (35.73 %) and non-steroidal anti-inflammatory drugs (15.93 %), Carbamazepine (18.25 %), phenytoin (13.37 %), fluoroquinolones (8.48 %) and paracetamol (6.17 %) (Patel et al., 2013[14]). Co-amoxiclav is a generally well tolerated antimicrobial. Its most frequently reported adverse effects are gastrointestinal adverse reactions and hepatotoxicity (Salvo et al., 2007[16]). It has been reported in few publications as etiology of SJS in adults (Abou-Elhamd, 2009[1]). Our patient did not report any of these. 

Amoxycillin and clavulanic acid combination therapy was identified as the causative agent because of the temporal relationship between the administration of the combination and the beginning of the eruptions. There have also been several other previous reports linking amoxycillin and clavulanic acid to Stevens-Johnson syndrome. According to Naranjo Adverse Drug Reaction Probability Scale, amoxycillin and clavulanic acid induced SJS was possible in our patient (a score of 4). The first step in the management was an immediate withdrawal of the offending agent followed by supportive care. Garcia-Doval et al. (2000[9]) report that earlier the drug is withdrawn, better the prognosis while exposure to drugs with longer half-lives increases the risk of death. Supportive care must include the management of fluid and electrolyte requirements (Garcia-Doval et al., 2000[9]). Adjuvant treatments such as corticosteroids and immunosuppressants may also be used in severe cases of SJS (Gerull et al., 2011[10]).

## Conclusions

This case report reports the fact that severe hypersensitivity reactions can occur with amoxycillin and clavulanic acid, which can be possibly dangerous and life-threatening. Therefore, clinicians must be more cautious while prescribing this drug. Affected patients and their first-degree relatives should be educated regarding the adverse effects of beta lactam antimicrobials and instructed to avoid them in future.

## Financial interests

None declared. 

## Conflicts of interest

The authors declare that they have no conflict of interest.
